# Moral reasoning through the eyes of persons with behavioral variant frontotemporal dementia

**DOI:** 10.3389/fneur.2023.1197213

**Published:** 2023-07-10

**Authors:** Rea Antoniou, Tobias Hausermann, Alissa Bernstein Sideman, Kristina Celeste Fong, Patrick Callahan, Bruce L. Miller, Joel H. Kramer, Winston Chiong, Katherine P. Rankin

**Affiliations:** ^1^Memory and Aging Center, Department of Neurology, University of California San Francisco, San Francisco, CA, United States; ^2^Philip R. Lee Institute for Health Policy Studies, University of California San Francisco, San Francisco, CA, United States; ^3^Department of Humanities and Social Sciences, University of California San Francisco, San Francisco, CA, United States; ^4^Global Brain Health Institute, University of California San Francisco, San Francisco, CA, United States

**Keywords:** moral reasoning, bvFTD, positive emotionality, mixed method approach, prosocial values

## Abstract

**Introduction:**

Persons with behavioral variant frontotemporal dementia (bvFTD) can exhibit apparently antisocial behaviors. An example is their tendency to adopt utilitarian choices in sacrificial moral dilemmas, i.e. harmful actions to promote overall welfare. Moral cognition models interpret such tendencies as deriving from a lack of emotional engagement and selective impairment in prosocial sentiments.

**Methods:**

We applied a qualitative approach to test those theoretical assumptions and to further explore the emotional experiences and values of people with bvFTD while they contemplate moral scenarios. We conducted semistructured interviews with 14 participants: 7 persons with bvFTD and 7 older healthy controls. Transcripts were coded using ATLAS.ti 5.0.

**Results:**

During the moral reasoning task, persons with bvFTD reported more positive emotions than negative and showed significantly less cognitive precision in their moral reasoning compared to controls. Persons with bvFTD also organized their choices predominantly around kindness and altruism, and their responses reflected higher rule compliance. Our study showed that bvFTD persons’ utilitarian responses to moral dilemmas did not arise from an emotionally disengaged or antisocial perspective. Instead, they were underpinned by positive emotionality and prosocial values.

**Discussion:**

These findings enrich current understandings of moral cognition and highlight the importance of incorporating mixed methods approaches in dementia research that take into consideration the viewpoint of cognitively impaired individuals

## 1. Introduction

Moral judgments engage moral cognition, operationalized as the application of cognitive processes to issues we identify as involving “right” and “wrong” behavior (1). Accordingly, models of moral cognition, such as the moral emotion and dual process theory (2, 3), have been developed to explain the cognitive underpinnings of moral judgments and tested in neurological conditions, with a view to determining whether they still hold true in individuals where specific aspects of cognitive processing are impaired. One such condition is the behavioral variant of frontotemporal dementia (bvFTD), a neurodegenerative condition predominantly affecting socioemotional function and, as a result, moral reasoning (4–6). In previous studies, persons with bvFTD have been characterized as individuals who, as opposed to healthy controls, are more inclined to endorse instrumental harm choices so that the greater good is obtained (e.g., being willing to push someone to their death to save five people), a type of choice referred to as “utilitarian.” (6, 7) Only a minority of healthy individuals in the population make these choices in moral reasoning scenarios. This expectation that persons with bvFTD will have an abnormally utilitarian outlook works in tandem with a conceptualization of bvFTD as a condition that encompasses a lack of social propriety that is motivated by antisocial traits. Those findings, however, derive from an empirical framework that studies moral reasoning quantitatively and excludes dilemmas that include a dimensional understanding of utilitarianism, where scenarios account for both impartial beneficence (i.e., benefitting strangers equally to close friends and family) and instrumental harm (8). This study aimed to draw a more nuanced picture of moral reasoning in bvFTD via a qualitative approach and the use of dilemmas that examine utilitarianism multi-dimensionally.

Emotions play a major role in the majority of our moral decisions, generating significant tilts to our moral compasses. As proposed by the moral emotion theory, for instance, emotions act as salience messengers to help us distinguish right from wrong, point to moral violations, and motivate moral behavior (2, 9). Because of their associated tone, those “moral emotions” are characterized as self-conscious (e.g., shame, guilt, and pride) and serve as emotional barometers by offering salient feedback on social and moral acceptability (10–12). For example, when individuals do the right thing, positive feelings of pride and self-approval are likely to emerge. Neurological accounts of bvFTD suggest significant impairment within the systems involved in the processing of moral emotions (i.e., medial, orbitofrontal cortex, and superior temporal cortices) (11, 13, 14). As a behavioral sequela, the engagement of prosocial sentiments is hindered. Based on that empirical framework, persons with bvFTD are thought to adopt anti-social decisions and behaviors due to the associated dysfunction of systems subtending moral emotions.

Theoretical accounts that support the role of emotions in moral reasoning are also found in dual-process models (3). While the moral emotion theory focuses on the specific contribution of moral emotions in tagging moral salience, the dual process model highlights two types of processes that subtend decision-making (15). More specifically, the dual-process theory of moral cognition asserts that moral decisions are the product of either one of two distinct mental processes (1, 3, 16): (1) automatic-emotional processes, which are deemed fast and unconscious and promote intuitive judgments (system 1) and (2) conscious-controlled processes, which are slow and deliberative (system 2). Moral decisions drawing from system 2 are less influenced by the immediate emotional features of decision-making (system 1). Instead, they seem to emanate from general knowledge and abstract moral concepts, often accompanied by a more controlled analysis of situational features. Characteristically it is suggested (15) that non-utilitarian judgments are mostly driven by automatic emotional responses, while utilitarian judgments engage controlled cognitive processes.

As emotional disturbances are a cardinal feature of bvFTD, most studies that examine moral reasoning in bvFTD and related neurological conditions contextualize their findings under the prism of dual process models, emphasizing system 1, namely the affective contributions that generate typical moral decisions (17). For example, it has been suggested that when persons with bvFTD, as opposed to healthy controls, are more likely to respond to sacrificial dilemmas by endorsing instrumental harm choices for overall welfare (i.e., utilitarian choices), it is a reflection of their diminished capacity for emotional response (6, 7, 18–20). Similarly, other types of behaviors that are generally deemed immoral (e.g., loss of social tact, unacceptable physical contact, improper verbal or nonverbal communication) are also interpreted under this affective framework. More specifically, the behaviors’ occurrence has been attributed to a diminished internal emotional experience, deficits in socioemotional attention, and decreased autonomic responsiveness (21–24).

Earlier studies indicate that the specific mechanism accounting for impairments in emotionally-based moral behavior is a decreased activation of one’s representations of the state and situation of others (19, 25). Individuals with bvFTD, for instance, do not seem to understand or embody the mental and emotional state of others due to diminished ability to simulate the same emotional state in themselves. In the case of sacrificial (or “personal”) moral dilemmas, a cognitively healthy individual would engage in harm-averse behavior to avoid the negative mental and emotional states brought on by pro-sacrifice responses. With bvFTD, however, the inability to embody and understand these same states may lead to more utilitarian responses, where individuals are more likely to accept harm as a means to promote the greater good. Thus, this lack of embodiment takes the form of an inability to represent the mental ramifications (i.e., the aversive internal experience of employing harm) brought on by adopting pro-sacrifice responses.

However, these findings are puzzling both on a conceptual and methodological level. First, utilitarian decisions are thought to reflect care and concern for the good of all sentient beings (26). Indeed, utilitarianism has been coined the “greatest happiness principle,” holding that actions are “right” to the extent that they promote happiness for the greatest number of people. Yet, a growing body of research has begun to link these very same ‘utilitarian’ judgments to antisocial traits like psychopathy and reduced empathic concern (22–24, 27). Hence the connotation of “immorality” associated with utilitarian choices in discussion of persons with bvFTD stands in sharp antithesis to this tenet (i.e., greatest happiness principle). In reviewing the literature on moral cognition, one can see how studies heavily rely on employing moral dilemmas that exclusively measure the instrumental harm tendencies of utilitarianism (i.e., the negative dimension). This might contribute to the characterization of persons with bvFTD as immoral. Impartial and altruistic tendencies (i.e., the positive dimension), in turn, often fall outside of the empirical scope of these studies (8).

Additionally, contemporary studies of moral cognition in bvFTD have tended to frame their understanding of moral reasoning by studying moral dilemmas in a way that generates quantitative outputs, i.e., proportions of utilitarian choices. Such an approach, while arguably permitting more objective understandings of moral cognition, disregards the rich information inherent in associated reasoning and psychological processes (e.g., emotional and cognitive elicitation) that subtend moral choices. In addition, studies rarely collect insights into values and perceptions of rules that could assist in contextualizing morality. This study was designed to bridge these methodological and conceptual gaps by examining moral reasoning qualitatively in healthy older individuals and persons with bvFTD. As such, we examined the psychological processes in moral reasoning through a phenomenological lens, and qualitatively describe morality through the eyes of the individual.

## 2. Materials and methods

### 2.1. Study design

This study used a mixed-method design, collecting quantitative (e.g., Mini-Mental State Examination) and qualitative data. The qualitative analysis included both pre-specified (deductive) and emergent (inductive) research questions (28, 29). On the one hand, an inductive design was employed so that participants’ responses to moral dilemmas could help generate new, emerging theoretical concepts and patterns (30). On the other hand, deductive approaches served to test theories derived from moral cognition models, and more specifically, to examine the presence of emotion elicitation and prosocial sentiments during moral reasoning.

### 2.2. Participants

All research participants were recruited at the Memory and Aging Center in the Department of Neurology, UCSF. We utilized purposeful sampling whereby participants were selected explicitly because of an existing bvFTD diagnosis (*N* = 7), i.e., met the clinical diagnostic criteria for probable bvFTD (4). For comparison and interpretation of the study results, we recruited healthy controls (*N* = 7), consisting of participants without any type of dementia diagnosis. The healthy control sample was drawn from community-dwelling older adults enrolled in a longitudinal study of healthy brain aging at UCSF. Participants in this cohort were verified as neurologically normal following a multidisciplinary assessment including (i) a neurological examination, (ii) in-person neuropsychological testing, and (iii) and an informant interview. A group of multidisciplinary professionals, including neurologists, neuropsychologists, and nurses established individual diagnoses in both the bvFTD and healthy control groups employing neuroimaging, neurological, neuropsychological, and behavioral assessments.

The Committee on Human Research at UCSF approved this study. Before testing, all participants signed informed consent forms, confirming voluntary research participation, and gave permission to use the collected data.

### 2.3. Moral reasoning task

Participants underwent a semi-structured interview with an embedded moral reasoning task consisting of seven moral dilemmas (see Supplementary Tables S1, S2). Three moral categories were tested, accounting for both positive and negative dimensions of utilitarianism.

#### 2.3.1. Categories falling under the Positive Dimension of Utilitarianism

1. Special obligations dilemmas are composed of three items concerning choices that assess one’s attitude toward favoring close others (e.g., family members, friends) at the cost of the greatest expected welfare. An example would be parental choices that prioritize one’s own child’s well-being over the well-being of other children. Here, utilitarian judgments reflect a disregard for tight social bonds in consideration of the greater good.
2. Agent-centered permissions dilemmas are composed of two items reflecting choices that assess one’s attitude toward improving others’ welfare at the cost of one’s own interests. An example would be whether to donate or keep the money for one’s personal use. In this case, the utilitarian choice disregards self-interest for the greater good.

#### 2.3.2. Category falling under the Negative Dimension of Utilitarianism

1. Personal rights dilemmas are composed of two items concerning choices that substantially affect the interests of other people, and in which the best overall outcome could only be produced by violating an individual’s rights. For instance, whether to push one person into the path of a runaway trolley that would otherwise kill five people. Here, the utilitarian choice is to sacrifice the individual, so that the greatest welfare is produced.

Additionally, we collected quantitative performance metrics (Likert scale 1–4, definitely/probably, yes/no), with lower scores reflecting higher utilitarian reasoning (max = 28). Additional quantitative scores were calculated for the three moral categories tested: special obligations (SO, max = 12), agent-centered permissions (AP, max = 8) personal rights (PR, max = 8).

### 2.4. Procedure

#### 2.4.1. Semi-structured interview

A semi-structured interview guide was developed to allow participants to describe their emotions, perceptions, and values as well as their underlying reasoning behind their responses to moral dilemmas (see Supplementary Tables S1, S2). The interview was divided into two main sections: (a) seven moral dilemmas with the option to respond in a utilitarian or non-utilitarian manner on a scale of 1–4 (probably/definitely – yes/no), Two follow-up questions exploring underlying reasoning (“Could you please explain in 2 to 3 sentences why you chose this option?”) and emotions involved in each moral decision (“How did you feel when responding to the dilemma?”) and (b) Questions contextualizing morality. Participants were asked to provide a wider and deeper understanding of their emotional and cognitive processing by characterizing their experience during moral reasoning. This section of the interview incorporated probe questions about values, rule compliance, and moral flexibility (e.g., Which is the most important value you try to live by?).

#### 2.4.2. Interviewer and interview procedure

The same interviewer (RA) performed all the interviews. Training for the interviews included work with a medical anthropologist (ABS) and sociologist (TH), reading qualitative interviewing technique books (30) and conducting five pilot interviews, followed by minor refinements to the interview guide based on participants’ feedback. The interviewer had psychological training with extensive experience working with this older age group. Interviews with the participants were scheduled after obtaining consent and permission to record. Each interview lasted approximately 40 min and was conducted online, via video conferencing software. It began with a presentation of the general scope of the study, which was portrayed as an invitation to discuss the moral dilemmas. Participants were instructed to provide their initial responses and the interviewer emphasized that there were no expected right and wrong answers. Participants could withdraw from the study at any time.

#### 2.4.3. Qualitative data analysis

Recorded interviews were sent for transcription. Each interview was then reviewed in its entirety against the verbatim transcription, with edits (e.g., corrections and additions) made where necessary. All transcriptions were deidentified. Each transcribed interview received multiple readings to obtain an understanding of each participant’s responses. Deductive codes were established according to the purpose of our study (e.g., emotion elicitation), but the codebook allowed for the identification of additional, inductive codes to note concepts that emerged during data review. Lower-order themes (e.g., joy) were used to form the broader scope of higher-order themes (e.g., positive emotion). In an iterative process, themes were refined and checked against the transcripts. Cross-evaluation of the themes was conducted with the consultation of the research team. Analysis was performed via ATLAS.ti software (30, 31).

#### 2.4.4. Rigor

To improve the rigor of the qualitative data analysis process, several validation methods were employed. This included, first, the documentation of the analysis procedure (e.g., codebooks and emerging themes described in detail). Second, regular research team meetings were held during the data collection and analysis process, to provide ongoing transparency and cross-validation of themes. Third, to reduce researcher subjectivity bias, we used the technique of triangulation. Five investigators coded the data independently and subsequently compared and discussed the codes until a consensus was reached. Lastly, divergences in theme categorization were discussed until a consensus was established (32).

## 3. Results

### 3.1. Participants’ characteristics

The participant sample (*N* = 14) was predominantly white (79%) and consisted of individuals ranging from 52 to 87 years old (M = 68.4 years, SD = 10.4). Participants’ general cognitive capacity, functional capacity, and mood were evaluated with a screening battery consisting of the Mini-Mental State Examination (MMSE), the Clinical Dementia Rating (CDR), and the Geriatric Depression Scale (GDS) respectively (see Table 1). For the bvFTD group, we used the CDR plus Behavior and Language domains from the NACC FTLD Module (CDR plus NACC FTLD) as a proxy of disease severity. The measure is an extension of the standard CDR and includes two additional domains that are predominantly affected in FTD: behavior and language. Each patient’s CDR plus NACC FTLD global score was calculated based on the scoring rules by Miyagawa and colleagues (32).

**Table 1:** Participant’s general characteristics.

Variables	bvFTD (*n* = 7)	HC (*n* = 7)
Demographics		
Age	63.1 (6.9)	73.7 (9.9)
Education	16.4 (2.9)	17.7 (1.8)
Gender		
Male/Female	4/3	4/3
General functioning		
Mini-mental state examination	24.5 (3.5)	29.3 (0.5)
Clinical Dementia Rating plus NACC FTLD		
Box score	7.2 (3.4)	0 (0)
Global score	1.4 (0.6)	0 (0)
Geriatric depression scale	9.7 (7)	2.7 (2.1)
Moral reasoning		
Special obligations	7.6 (1.5)	7.6 (2.6)
Personal rights	4.3 (1.7)	4.9 (1.7)
Agent-centered permissions	3.3 (1)	3.3 (0.5)
Overall utilitarian	15.1 (2.4)	15.7 (3.6)

Values represent mean (SD), except gender which represents frequency.

Persons with bvFTD’s general performance revealed mild impairment in overall cognitive functioning (MMSE; M = 24.5, SD = 3.5), while the average CDR plus NACC FTLD total score was 1.4, indicating that this sample represented the earliest stages of disease progression, at a “mild dementia” level. Scores on GDS suggested significantly more depressive symptoms in the bvFTD group (M = 9.7, SD = 7) than in the healthy controls (M = 2.7, SD = 2.1), though on average the group was below the screening threshold for mild depression on this measure (i.e., scores above 13).

In terms of moral reasoning, the healthy control (M = 15.7, SD = 3.6) and the bvFTD (M = 15.1, SD = 2.4) groups’ average overall utilitarian scores reflected diverse responses that encompassed both utilitarian and non-utilitarian choices, as both groups scored in the middle range of utilitarian performance. Characteristically, both groups chose more utilitarian responses in the agent-centered permissions moral category, followed by the personal rights and special obligations moral category (see Table 1). In qualitative data analysis, 238 primary codes were extracted and classified as broader themes of emotion elicitation, cognitive elicitation, and contextualization of morality.

### 3.2. Theme 1: emotion elicitation during moral reasoning

Participants’ reasoning across moral dilemmas involved emotion elicitation, revealed by answers to the question following each dilemma: “How did you feel when responding to this dilemma?.” Healthy controls and persons with bvFTD were characterized by a different emotional palette, revealing a between-group distinction on the valence of the emotion they reported experiencing while considering their moral responses. Positive emotions, particularly joy were more prevalent when persons with bvFTD contemplated their feelings associated with their reasoning (82% of all joy responses), as opposed to healthy controls, who tended to express more negative emotions, particularly guilt (79% of all guilt responses; see Table 2).

**Table 2:** Across-group comparisons of emotion elicitation during moral reasoning.

Emotion elicitation	bvFTD (*n* = 7)		HC (*n* = 7)	
	Individuals No (identity)	# of occurrences	Individuals No (identity)	# of occurrences
Negative emotions				
Frustration	2 (1/7)	4 (36%)	4 (3/4/6/7)	7 (64%)
Sadness	4 (1/5/6/7)	12 (44%)	6 (2/3/4/5/6/7)	15 (56%)
Guilt	1 (1)	1 (21%)	3 (2/4/7)	5 (79%)
Positive emotions				
Relief	1 (5)	1 (56%)	1 (5)	1 (44%)
Joy	4 (1/2/6/7)	10 (82%)	2 (6/7)	2 (18%)
Pride	3 (2/5/7)	4 (37%)	4 (1/2/4/5)	7 (63%)

Absolute frequencies and proportions after normalization.

Although emotional blunting constitutes a core diagnostic feature of bvFTD, participants still described feeling an emotional response when the interviewer probed for a description, and some described their response with some emphasis. For instance, when asked whether they would refuse to take money for personal use, to instead donate it to cure HIV, one participant responded:

*I feel good that I was able to say I do not need the money. I feel good that somebody else is going to do good because I did not take their money. [bvFTD No. 7].*

The same participant – in addition to reflecting on the joy associated with donating money for a good cause – emphasized the positive felt experience of responding in a utilitarian way. They added:

*It’s a good feeling inside. [bvFTD No. 7].*

Along with their felt emotional experience, 5 persons with bvFTD also mentioned the ease associated with the decision-making process. One participant, when reasoning about whether they would undergo repeated blood donations to keep a person from dying, stated:

*It’s, you know, fantastic to save a life. You know and it means nothing to me. I felt great, it was no brainer. [bvFTD No. 2].*

Some participants with bvFTD also expressed a sense of empowerment associated with their utilitarian decisions, as though a sense of self-approval was associated with making the decision, which added to the positive feelings they described. One participant’s utilitarian decision (specifically, to personally approve the removal of one’s man kidney in order to help a vitamin-deficient family to survive) was accompanied by:

*I guess I feel empowered to do that. I would feel like, what would be another word? You want one of those feelings words, right? [Interviewer: What do you mean by feeling empowered?] I guess I feel I have the right to make that decision if I had to make that decision. [bvFTD No. 5].*

This more positively valenced emotional outlook stood in antithesis to the healthy controls, more of whom expressed negative emotions associated with their moral responses, sometimes very strongly. Reflecting on their feelings associated with the dilemma of whether they would sacrifice their nephew to save 6 people, healthy-control participants replied:

*Yes, well there’s sadness. There’s sadness. There’s some frustration because the kid cannot swim. But it’s primarily sadness for the fact again of the loss of life. [HC No. 6].*

*Horrified. Absolutely completely horrified. Devastated. Horrified. Super upset. Just horror-stricken. Sort of in shock. Probably would not even feel how truly sad I felt for a while until the shock wore off. It was just absolutely horrible. [HC No. 7].*

When responding to the same dilemma (i.e., sacrificing the nephew for the six strangers) some healthy controls were even able to tap into the bodily dimension of the expressed emotion, articulating visceral sensations, an occurrence that did not explicitly occur in the bvFTD sample. As one participant put it:

*My stomach is turning inside out. That’s my feelings. [HC No. 5].*

In the healthy control group, the majority of participants expressed sadness, frustration, and pride, with three participants expressing guilt, two expressing joy, and one relief as the core emotions accompanying their moral reasoning. Those emotions were often expressed in a more complex, layered manner. Also, self-conscious emotions including guilt and pride were more often expressed by individuals in the healthy control group, while simpler emotions such as joy were less common in this group than in the individuals with bvFTD. This suggests the availability of a more nuanced emotional palette when reflecting on their feelings about their moral responses. Following their response that they would not endure repeated blood donations to prevent a person from dying, one participant described their feelings as follows:

*Sad and worried about him. And, you know, sort of regretful that I did not feel like I could help him. I feel like a desire to help him and a wish, or her, whoever it is, person. Is it a man? Anyway, whomever it is. A desire to help. And I would feel regretful that I wasn’t choosing to do it. I would feel a loss. A real sadness and regret. Very sad. [HC No. 7].*

### 3.3. Theme 2: cognitive elicitation during moral reasoning

When contrasting moral reasoning across persons with bvFTD and healthy controls, participants’ responses involved a variety of cognitive processes, which we included as an additional core theme of cognitive elicitation (see Table 3). The types of cognitive responses observed appeared to reflect four major categories, two of which reflected positive dimensions (i.e., projection/imagination and insight), and two of which reflected weaknesses in cognitive processing (i.e., cognitive imprecision and poor elaboration). Each of these cognitive elicitation categories differed between the bvFTD and healthy individuals, with responses reflecting projection (82%) and insight (91%) being more common in healthy controls, and responses reflecting cognitive imprecision (67%) and poor elaboration (91%) being more common in persons with bvFTD.

**Table 3:** Across-group comparisons of cognitive elicitation during moral reasoning.

Cognitive elicitation	bvFTD (*n* = 7)		HC (*n* = 7)	
	Individuals No (identity)	# of occurrences	Individuals No (identity)	# of occurrences
Projection/Imagination	4 (1/5/6/7)	7 (18%)	7 (1/2/3/4/5/6/7)	32 (82%)
Insight	1 (7)	1 (9%)	3 (1/2/5)	10 (91%)
Cognitive imprecision	6 (1/2/3/4/6/7)	14 (67%)	1 (4)	7 (33%)
Poor elaboration	5 (1/2/3/4/6)	30 (91%)	2 (6/7)	3 (9%)

Absolute frequencies and proportions after normalization.

Four individuals in the bvFTD group engaged in projection or imagination, compared to all seven of the healthy controls, and this cognitive process appeared much less frequently in their responses. Specifically, we defined the cognitive process of projection/imagination as the ability to put oneself into the moral scenario as if one was the main character of the dilemma, more richly taking the perspective of the protagonist or recipient by imagining one is giving or experiencing the harms or benefits personally. Given that this cognitive process was seen in 100% of healthy controls, this seems to be an important element of healthy moral reasoning, perhaps providing a deeper experience of the dilemma through the felt experience of its ramifications.

For example, concerning the dilemma of whether they would give the medicine to their own child rather than another child, one participant with bvFTD showed projection in the following manner:

*Because I would like to help people, but I have [unintelligible thoughts] children and I want to be with them. And if it means I’m going to die, I mean … [bvFTD No. 6].*

*I feel that my … I’m about to. I feel that if I chose to sacrifice six people for the life of one, even though I have a relation to that one, is a selfish thing to do. When in fact, those six people have other people that feel for them. So, why take my feelings out of the equation, it’s better to save more than less. [bvFTD No. 7].*

Healthy controls often embedded such cognitive processes in their moral responses. In response to the same dilemma, one participant responded:

*One of my rules as a parent is to protect my child. So, if there’s a choice between my child and another child, I’m always going to pick my child. [HC No. 1].*

In another dilemma, where the participant was asked whether they would save their nephew instead of six strangers, they answered:

*I mean, I kind of put myself in the place of, like, thinking of it as nephew or even your son, like, also, you know, it’s a family member, you know, you’d have to explain it to their family at some point, so – but you would hope that, like, you know, there’s six other people that lived, you know, that went on to do their lives. So, yeah, again, it’s hard to do it purely on math, but, you know, it’s not that strangers have any less value than my nephew. [HC No. 2].*

The degree of insight and awareness underlying the participant’s moral responses also differed between the two groups, though this cognitive tendency was seen in fewer participant responses overall (4/14). Only one person with bvFTD expressed metacognitive insight in only one response, while 3 of the healthy controls showed insight on 10 occasions. Healthy controls seemed able to track more accurately their thought and emotional processes accompanying their feelings and reasoning towards moral dilemmas. On the footbridge dilemma, a participant contemplated the reasons for opting to not push a man off the bridge to save the five workers in this way:

*It’s a tough choice. But I went with the first reaction, to the first emotional reaction that I had. I went with that. Because I could sit here for two or three minutes and change my mind back and forth probably. But I think the real true response came immediately for me. And that was I’m not going to push the guy off the footbridge. [HC No.1].*

Metacognitive insight was also observed in the bodily realm. In the same dilemma, a participant expressed insight into how their mind and body could function as a moral compass for their moral judgments:

*Because – as I said because it would be – I would find it very hard to take affirmative action to do something which causes someone to die. It’s much easier – and I think – as I said, I think I would be paralyzed, probably, out of, you know, the god awfulness of the situation to do anything. And there’s just – I just do not think that I could bring myself to go through – to do that. I do not know if it’s a moral dilemma. I think it’s more just my mind and body would probably stop me from doing anything – anything, you know, at all. [HC No. 5].*

Another category of cognitive elicitation observed in participant’s responses was termed “cognitive imprecision,” to describe participants’ tendency to reconstruct the premise of the dilemma and fail to approach the dilemma according to its internal logic, showing an underlying resistance to the scenario’s structure, and breaking the contractual rules of the posed dilemma. In our sample, the large majority of persons with bvFTD (6 of 7) showed this tendency, compared to only 1 of the healthy controls. Alternating the structure of dilemmas seemed to facilitate decisions and make it easier for participants to respond when the dilemma posed a difficult conflict. For instance, when asked to answer the dilemma that involved pushing a stranger onto the tracks to save five workers, some participants resisted the structure of the dilemma requiring the death of the five workers:

*Well, even though the way it’s worded sounds pretty positive, the death of the five workers is not absolutely known. Maybe they’ll look up right away and jump. You know, there are possibilities there, but the – the large person that’s right beside me, his – his life is known, and I’d be, you know, sort of the bird in the hand versus the bird in the bush. [bvFTD No.1].*

*I just think it’s wrong to do something bad to make something good happen. I think it would be better, for example, for me to jump out there to save all of them. [bvFTD No. 4].*

Similarly, another participant, responding to the dilemma about whether they would give a medicine to their own child or another child, when the medicine was explicitly described as ineffective if the dose was shared, remarked:

*Well, I’d probably try to split it. [bvFTD No.3].*

An additional category of cognitive elicitation observed in our sample was termed poor elaboration, which was operationalized as providing an inadequate explanation underlying participants’ reasoning despite maximal probing by the interviewer. Persons with bvFTD more often failed to elaborate on their responses (making 91% of poor elaborations, with 5 of the 7 bvFTD participants providing 30 poor elaborations, compared to 3 in the healthy controls). This response style was observed to reflect difficulty providing more in-depth, sophisticated reasoning about their moral choices. For example, when asked to contemplate their feelings about why they would agree to give repeated blood donations to save a person from dying, a participant answered:

*It’s always good to help people, but you know. [bvFTD No.6].*

By the same token, another participant when presented with the same dilemma, expressed their feelings with the following short answer:

*Well, it’s a conflict, again. [bvFTD No.3].*

### 3.4. Theme 3: contextualization of moral reasoning

One of the goals of this study was to also contextualize morality in terms of participants’ perception of values and rule compliance. For this reason, after responding to all of the moral dilemmas, participants were asked to openly reflect on the values that they try to live by and their perceptions about rule-breaking. We additionally coded whether participants adhere to philosophical and religious standards, including a frame of reference from which their values seem to emanate.

When asked about the most important value they try to live by, we found that some participants associated their moral reasoning with values reflecting suggesting the presence of a prosocial compass – fairness, kindness, honesty, and the greater good. This tendency spanned both study groups fairly evenly, though more statements of “kindness” as a value were seen in persons with bvFTD, while healthy controls were slightly more likely to espouse the value of fairness (see Table 4). Both groups were equally likely to endorse the greater good as a guiding value. For example, persons with bvFTD responded:

**Table 4:** Across-group comparisons of participants’ perception of values and rule.

Contextualization of moral reasoning	bvFTD (*n* = 7)		HC (*n* = 7)	
	Individuals No (identity)	# of occurrences	Individuals No (identity)	# of occurrences
Prosocial values				
Fairness	1	1 (29%)	3 (1/2/5)	3 (71%)
Kindness	3 (3/4/5)	4 (78%)	1 (7)	1 (22%)
Honesty	1 (7)	1 (38%)	2 (1/2)	2 (62%)
Greater good	2 (2/6)	2 (53%)	2 (3/4)	2 (47%)
Rule perception				
Rule breaking	2 (4/5)	3 (30%)	6 (2/3/4/5/6/7)	6 (70%)
Rule compliance	5 (1/2/3/6/7)	5 (82%)	1 (1)	1 (18%)
Philosophical and religion standards				
Adherence	2 (1/2)	3 (72%)	1 (5)	1 (28%)

Absolute frequencies and proportions after normalization.

*Try to be as helpful as I can. [bvFTD No.3].*

*I guess to love the person you are talking to at the time. [bvFTD No.4].*

*To give love and receive love from other people. [bvFTD No.5].*

The values of healthy controls, in turn, focused more on honesty and fairness. Asked to express which values they live by, participants gave the following responses:

*Honesty is one of them. Being true to myself is another one. [HC No1].*

*I think I try to be I want to be fair and treat everyone equally. That’s the most important value to me is that everybody – I do not know if you call that a value in the different types of values, but I guess equality, equal. [HC No2].*

*Do no harm. The golden rule. I mean, however – every different society has a different way of expressing it. But I just think it’s do unto others as they would – you would have them do unto you – or do not do to others what you would not like to have them do to you. Yeah. I mean that – the golden rule is – I would not say it’s at the front of my consciousness all the time. But I absolutely believe in it. [HC No5].*

Interestingly, while reflecting on their values, more of the persons with bvFTD seemed to base their prosocial compass on philosophical and religious standards than healthy controls (see Table 4). This observation highlighted an external frame of reference around which persons with bvFTD may have constellated their moral beliefs:

*Well, I think the most important value is I tried to depend on the Lord to help me with things. Sometimes I forget, but real closely related to that is I try to do unto others as – as I think I would feel good if they were doing unto me. That’s not necessarily as – as they would treat me but as I would like to have them treat me. But most of them can be related to God. Is that too nebulous? [bvFTD No1].*

*I live by a code, you know, it’s the marine code. It’s great! it makes me feel alive. Serve and protect, the people of the world [bvFTD No2].*

In terms of rule compliance, a sharp differentiation between healthy controls and persons with bvFTD prevailed. Persons with bvFTD were more rule compliant in their responses, emphasizing the expression of moral facts when dealing with breaking the rules. For example, when asked whether they would break a rule, bvFTD persons were more likely to give broad negations that were lacking in situational nuance, responding:

*I spent a lot of years as a principal and a teacher and, no, I guess the answer is no. I just would not do it. I would not break a rule in general. [bvFTD No1].*

*No, I would not. no, I would not. Because it’s the wrong thing to do. It’s totally wrong. [bvFTD No2].*

*I do not think you should. [bvFTD No 3].*

*No. It’s not good to break rules. [bvFTD No7].*

On the contrary, healthy controls seemed more willing to break a rule to achieve a goal, reflecting moral flexibility and a tendency to contextualize their behavior. As three participants stated:

*Yeah. So, for example, in a lot of times in, like, building processes and things that we are doing now, and a lot of the building municipalities just take forever, and there’s a lot of red tape and a lot of bureaucracy, and I will not break a rule, per se, but I’ll omit steps to get to it, again, if I know the goal is a good one and we had discussions, it’s not my own personal goal, yeah, I would do break a rule. I’m not a rule breaker just to do it. If it’s around safety for the most part I would not, but, yeah, I would do it in instances. [HC No2].*

*If the goal is to help someone, that’s what I was thinking. Yes. Oh, my. But in another situation, if the goal is my goal, you know, I want to win; therefore, I’m going to break a rule, no, I would not do it. But if it’s to help someone, yeah, I would break the rule. [HC No 3].*

*Well, there’s really no need, no reason not to, and as I get older and as you get older, even in good health you start to contemplate your death, and there’s too much that has to happen in the world for me to be satisfied, and I do not like the direction we are going away from what I consider to be necessary. My goals are being more and more ignored and made improbable. And to achieve that goal I would break a law. If I would not get caught. [HC No4].*

## 4. Discussion

This research is one of the few studies to directly reveal the voices of persons with bvFTD by giving them the opportunity to voice their reasoning, feelings, and values when responding to moral dilemmas. In previous studies, bvFTD has been associated with impairment in socioemotional function, which has been presumed to be the primary reason they are more likely to make utilitarian choices in sacrificial moral dilemmas in which they are willing to endorse harm in service of the greater good. However, our study captured a more holistic understanding of their thought processes by examining moral reasoning qualitatively, and by using a more nuanced measure of utilitarianism. This revealed that some of their utilitarian responses could be characterized as prosocial choices where they sacrificed their own self-interest to support the greater good. Persons with bvFTD were more likely than has previously been reported to endorse prosocial values such as kindness and service to the greater good as important personal values contextualizing their moral choices, and they exhibited significant positive emotionality around even difficult moral decisions.

Our research identified three core themes. The first represents participants’ responses to the question, “How did you feel when responding to this moral dilemma?” and thus captures the emerging emotions during the moral reasoning process. In line with previous literature (7, 33), in our study responses of persons with bvFTD were characterized by positive emotionality (e.g., *“I felt great.”*), whereas healthy controls were more likely to tag their moral reflections with negative emotions (e.g., *“I felt sad and worried.”*). Disrupted experience of emotion, particularly in relation to self-conscious moral emotions such as guilt (12, 17, 34), might account for the different emotional responses between persons with bvFTD and healthy controls. In previous research, this diminishment of self-conscious emotional responses in bvFTD has been predominantly understood in a negative light because it may lead to inappropriate behavior that can be detrimental to their social milieu. In the context of moral reasoning, this lack of self-conscious emotion takes the form of exhibiting not only a lack of guilt but also more positive emotionality when endorsing decisions where harm must be inflicted to ensure the overall welfare of a group or community. Interestingly, we found that in bvFTD prosacrifice and altruistic/impartial choices were accompanied by a more undiluted experience of positive emotion, which participants associated with “doing the right thing.” Thus, our study suggests that decreased sensitivity to guilt may be the cornerstone of the overall capacity to be impartially concerned for the greater good (i.e., whether or not some harm infliction is required to achieve overall welfare).

The suggestion that impaired socioemotional function may not hinder, but rather facilitate moral decision-making, has previously been introduced (16, 35). Prinz, for instance, argues philosophically that in certain situations, emotional processes such as affective empathy may actually pose a risk to moral judgment, suggesting that empathy can even have a detrimental impact on the ability to adopt moral judgments that promote overall welfare over the interests of close others. While much attention has been devoted to highlighting the negative aspects of utilitarianism, specifically the inclination to endorse instrumental harm in the context of bvFTD, our findings bring forth a different perspective. Significantly, our research illustrates how impartial tendencies can potentially be attributed to reduced socioemotional responsiveness, as indicated by diminished guilt and a potential lack of affective empathy. This shifts the focus from solely emphasizing negative aspects of loss of empathy to recognizing the underlying mechanisms that contribute to these tendencies and highlighting the retention of prosocial inclinations even in the context of empathy loss.

The second emergent theme, in which participants’ cognitive thought processes during moral reasoning were clarified, further elucidates the complex mechanisms embedded in moral reasoning. Our analysis of the responses of persons with bvFTD revealed a quality that we termed “cognitive imprecision.” In the context of moral reasoning, this appeared when participants resisted the given structure of the dilemmas, instead circumventing difficult decisions by changing the rules or resisting what the interviewer was asking them to decide (e.g., “*I think it would be better* … *for me to jump out there to save all of them*”). This observation is in line with previous literature, where this cognitive approach has previously been described in the context of bvFTD as *denkfaulheit* or “mental laziness,” (36) operationalized as an inappropriate cognitive shallowness characterized by a lack of depth and drive. In the participants with bvFTD in our study, this imprecision was compounded by poor elaboration, where participants often were unable to clarify or explain their moral reasoning when directly asked. By comparison, healthy controls demonstrated considerably greater cognitive precision and tolerance of nuance in their responses and were able to elaborate their responses more richly. Of note, future studies conjoining qualitative and quantitative measures of metacognition could further shed light on how metacognitive abilities influence moral reasoning in bvFTD and contribute to our overall comprehension of the cognitive processes involved in moral decision-making.

Our data illustrate how moral reasoning engages an interplay between psychological processes, in which emotional and cognitive disruption correlate, and may have important neural interdependencies. Neuroimaging studies of moral reasoning in persons with bvFTD have revealed that a primary source of divergence from healthy controls is altered activation of the salience network (SN) (19). This network is focally affected early in the bvFTD disease process and is pathognomonic to the disease, thus the alterations we observed in metacognitive insight, complex thinking, and elaboration in the bvFTD group, are most probably derived from salience network (SN) dysfunction. Chiong and colleagues (2013) showed that salience-driven attention mediated by the SN can act as a gating mechanism that influences the function of other brain networks and their associated cognitive processes (19). In the context of moral reasoning, when healthy individuals detect moral dilemmas as personally salient, the activation of the SN increases the likelihood of default mode network (DMN) engagement. As a result, autobiographical, self-referential, and perspective-taking processes are employed for more comprehensive and complex reasoning (25, 35, 37). Alternatively, when the SN does not alert the individual that a dilemma has personal relevance (either because it does not involve a personal moral component, or because of dysfunction in the SN), the individual is more likely to approach the dilemma in an impartial manner, activating the adaptive executive control (i.e., frontoparietal) network in the brain instead of the DMN. Our observation that 100% of healthy control participants employed imaginative perspective-taking while contemplating the dilemmas, but this was rarer in persons with bvFTD, is likely related to this lack of activation of the DMN that has resulted from altered SN function.

The third theme we identified in our data involved the contextualization of moral reasoning as a part of one’s overarching value system. In our sample, both persons with bvFTD and healthy controls espoused a number of prosocial values that guided their actions, including fairness, kindness, honesty, and the greater good. They also showed roughly equivalent adherence to philosophical and religious standards, though this was slightly greater in persons with bvFTD. Importantly, persons with bvFTD did not show antisocial or cold tendencies in their moral reflections when asked to reflect on their values, suggesting their motives were not as self-centered as implied by existing literature (18, 21, 22). However, we additionally observed the preservation of bvFTD participants’ perceptions regarding rules, with bvFTD participants providing many more responses centering around rule compliance than controls (e.g., *“rules should not be broken”*). Healthy controls, in turn, exhibited markedly more rule-breaking tendencies and moral flexibility in their responses (e.g., *“to achieve that goal I would break a law”*). Selective degeneration of neural systems important for mental flexibility could be associated with this increased rule adherence in the participants with bvFTD, and this finding may reflect some mental rigidity (38). Of note, our participants with bvFTD were more likely to actually break the rules of the dilemmas and to resist the interview structure in a cognitively imprecise manner, despite their explicit support of rule compliance when asked to describe their values.

Using a moral reasoning task that accounts for the two-dimensional nature of utilitarianism, along with probing questions regarding values, appeared to be instrumental to understanding moral cognition in bvFTD. For example, bvFTD and HC groups alike responded in a utilitarian way to some of the dilemmas, and this occurred across both personal rights dilemmas (negative dimension) and agent-centered permission and special obligations dilemmas (positive dimension). Importantly, our study challenges existing theories about moral reasoning in bvFTD that recognize utilitarian judgments as stemming from a lack of prosociality (17, 20, 21). Because their utilitarian tendencies span both positive and negative dimensions, and their endorsement of both prosocial values and positive emotions around doing what they believed was right, bvFTD participants’ choice to employ harm for the greater good did not appear to reflect a lack of impartial concern. Our findings echo other studies that identified the value of incorporating both positive and negative dimensions of utilitarianism (8, 37).

By leveraging a phenomenological approach, we demonstrate how emotional and cognitive processing interact in the service of moral reasoning. Based on our findings, we propose that knowledge of values and rules is preserved in bvFTD, and to some extent is conveyed in participants’ responses to moral dilemmas. One could assume that persons with bvFTD responded in a way that reflected the retention of a moral compass encapsulating both the dimensions of discipline (rule compliance) and prosociality (kindness). It remains of great interest to explore whether, and how, these findings vary across other dementia syndromes, such as Alzheimer’s disease syndrome and semantic variant primary progressive aphasia. Finally, despite not being the focus of our study, significant potential scope exists for examining additional processes involved in moral reasoning, such as empathic concern (39), faith (40), and sociocultural background.

Exploring the practical implications of these findings within the context of dementia care, particularly in therapeutics, is an important area for consideration. One notable application lies in the realm of enriching psychosocial interventions between individuals with bvFTD and those in their social environment, wherein cultivating symbolic meaning for careers assumes a central role (41). This enrichment is effectively operationalized through shared activities that serve as a foundation for fostering positive relationship gains. Our research might provide an example of ways to enrich communication by actively engaging persons with dementia to talk about their beliefs and values, and surfacing the prosocial views of individuals with bvFTD. Caregivers, in particular, stand to benefit significantly from this approach by reconnecting with the care recipients’ fundamental values, such as kindness, in a deeply meaningful manner. As a result, their caregiving experience may be profoundly reframed as more purposeful and fulfilling.

Our study has several limitations. Foremost, our study examines the responses of a small and somewhat homogenous sample. Participants’ responses are grounded on cultural norms and ways of life in the United States, and more specifically the West Coast. Thus, different perspectives and views could emerge from other cultural and research contexts. Expanding the sample size and broadening cultural variation would further elucidate the themes noted in this study and perhaps reveal additional factors. Additionally, our sample represents the earliest stages of bvFTD disease progression, whereas participants at a later stage might show different results. Lastly, this study relies on moral vignettes to measure moral reasoning instead of direct observations of real-life behavior. The dilemmas represent the participants’ ideas about hypothetical behaviors rather than observed occurrences. Even though we asked participants to report on their moral reasoning and describe their values, our design did not assess moral behavior in real life. Future studies might achieve greater ecological validity by comparing similar dilemmas to real-world moral behavior. Overall, these results more comprehensively reflect the emotional and cognitive experience of persons with bvFTD by centering their voices, and further highlighting the importance of incorporating qualitative approaches in dementia research.

## Data availability statement

Individual-level data presented in this article cannot be made available in a public repository because it consists of raw interview data of a sensitive nature and thus is subject to healthcare privacy regulations. Appropriate sharing of group-level or deidentified aspects of individual-level data will be possible upon request to https://memory.ucsf.edu.

## Ethics statement

The Committee on Human Research at UCSF approved this study. Before testing, all participants signed informed consent forms, confirming voluntary research participation, and gave permission to use the collected data.

## Author contributions

RA: conceptualization, writing–original draft preparation, resources, methodology, and writing–review and editing. TH and AS: methodology, investigation, resources, and writing–review and editing. KF and PC: methodology and writing–review and editing. BM and JK: resources and writing review and editing. WC: investigation, resources, and writing–review and editing. KR: conceptualization, resources, supervision, project administration, and writing–review and editing. All authors contributed to the article and approved the submitted version.

## Funding

This work was supported by the National Institutes of Health under grant numbers R01AG029577/RF1AG029577 (PI: Rankin), P01AG019724 (PI: Miller), NIAK01AG059840 (PI Bernstein), and the Larry L. Hillblom foundation under Grant [2014-A-004-NET (PI: Kramer)].

## Conflict of interest

The authors declare that the research was conducted in the absence of any commercial or financial relationships that could be construed as a potential conflict of interest.

## Publisher’s note

All claims expressed in this article are solely those of the authors and do not necessarily represent those of their affiliated organizations, or those of the publisher, the editors and the reviewers. Any product that may be evaluated in this article, or claim that may be made by its manufacturer, is not guaranteed or endorsed by the publisher.
