# Supplementary material for: Moral reasoning through the eyes of persons with behavioral variant frontotemporal dementia
Source: Front Neurol. 2023 Jul 10;14:1197213. doi: 10.3389/fneur.2023.1197213 (PMC10365271; doi:10.3389/fneur.2023.1197213)
Supplement: Supplementary file 1 [file Table_1.DOCX]

Supplementary Material

Moral Reasoning Through the Eyes of Persons with Behavioral Variant Frontotemporal Dementia

Rea Antoniou*, Tobias Hausermann, Alissa Bernstein Sideman, Kristina Celeste Fong, Patrick Callahan, Bruce L. Miller, Joel H. Kramer, Winston Chiong and Katherine P. Rankin

*** Correspondence:** Corresponding Author: Rea.Antoniou@ucsf.edu

# Supplementary Data Tables

**Table 1. Semi-structured Interview Part A: Moral Dilemmas by Category**

| Personal Rights | 1. Imagine that a runaway trolley is heading down the tracks toward five workers and will kill them if it keeps going. You are on a footbridge over the tracks, in between the approaching trolley and the five workers. Next to you on this footbridge is a stranger who is very large. The only way to save the lives of the five workers is to push this stranger off the bridge and onto the tracks below where his large body will stop the trolley. The stranger will die if you do this, but the five workers will be saved. Would you push the stranger onto the tracks to save the five workers?  *Possible Answers*   - Definitely yes = 1 - Probably yes = 2 - Probably no = 3 - Definitely no = 4   Could you please explain in 2 to 3 sentences why you chose this option? How did you feel when responding to this dilemma?  2. Imagine that you are leading a group that is lost in the wilderness. Your group includes a family of six with a genetic vitamin deficiency. A few people's kidneys contain large amounts of this vitamin. There is one such person in your group. The only way to save the lives of the six family members is to remove one of this man's kidneys and take the necessary vitamins from it. He will not die if you do this, but his health will get worse. He does not want to give his kidney, but you have the power to do what you choose. Would you take this man's kidney to save the vitamin-deficient family?  *Possible Answers*   - Definitely yes = 1 - Probably yes = 2 - Probably no = 3 - Definitely no = 4   Could you please explain in 2 to 3 sentences why you chose this option? How did you feel when responding to this dilemma? |
| --- | --- |
| Agent-centered Permissions | 1. Imagine that while waiting for a bus you begin to talk with a stranger. You have seen him on TV news shows, and he is a very rich but strange man. He says that he has $10,000 in his wallet, and then gives you a choice. He says you can take the money for personal use. However, if you do not take the money, he will donate $1,000,000 to a charity to cure HIV. He will not make that donation if you take his money now. Given what you know about him, you are sure that he will keep his word. Would you refuse to take the money so this man will donate more money to cure HIV?  *Possible Answers*   - Definitely yes = 1 - Probably yes = 2 - Probably no = 3 - Definitely no = 4   Could you please explain in 2 to 3 sentences why you chose this option? How did you feel when responding to this dilemma?  2. Imagine that you have a very rare type of blood. There are some people who can only receive blood transfusions from you, though you cannot receive blood from them. One of these people is a stranger who is very sick and will require many blood transfusions in order to survive. To donate all the blood that this person will need to get well, you will have to live in the hospital for six months. During this time you will undergo multiple large blood donations. These donations will make you feel weak and tired most of the time. Would you agree to these repeated blood donations to keep this person from dying?  *Possible Answers*   - Definitely yes = 1 - Probably yes = 2 - Probably no = 3 - Definitely no = 4   Could you please explain in 2 to 3 sentences why you chose this option? How did you feel when responding to this dilemma? |
| Special Obligations | 1. Imagine that you are driving a motorboat when a cruise ship nearby begins to sink. Looking left, you see that your nephew, who was on the ship, is holding on to a piece of floating wood. If you drive towards him at full speed you can save him right before he sinks. In the opposite direction is another piece of wood with six strangers clinging to it. By driving at full speed in the opposite direction, you have just enough time to save them. You only have enough time to rescue either your nephew or the six strangers. There are no other boats in the area. Would you save your nephew instead of the six strangers?  *Possible Answers*   - Definitely yes = 4 - Probably yes = 3 - Probably no = 2 - Definitely no = 1   Could you please explain in 2 to 3 sentences why you chose this option? How did you feel when responding to this dilemma?  2. Imagine that you are camping with your own small child and another child. Both children eat leaves from a poisonous plant. If untreated, your child will have painful boils all over his body for a year. The other child ate more leaves, and he will have painful boils for five years if he does not get treatment. Neither will have permanent scars or damage. You have a medicine that will keep these boils from appearing if a child drinks it immediately. Unfortunately, you only have one dose of this medicine. The medicine will not work if the dose is shared between the children. Would you give the medicine to your own child rather than the other child?  *Possible Answers*   - Definitely yes = 4 - Probably yes = 3 - Probably no = 2 - Definitely no = 1   Could you please explain in 2 to 3 sentences why you chose this option? How did you feel when responding to this dilemma?  3. Imagine that you are a doctor researching a bad infection for which there is no cure. You discover a mold that makes a medicine that cures this infection. Before you can tell others about your results, your own patient contracts the infection and will die without treatment. In order to save her you must use up all of your mold. It would take two years to grow another batch. During this time, a hundred other people will die of this infection. These people could be saved if you do not give the medicine to your patient and continue your research. Would you allow this patient to die in order to save many more lives in the future?  *Possible Answers*   - Definitely yes = 1 - Probably yes = 2 - Probably no = 3 - Definitely no = 4   Could you please explain in 2 to 3 sentences why you chose this option? How did you feel when responding to this dilemma? |

*Note:* This table contains the moral reasoning task categorized by moral category: Special Obligations dilemmas are composed of three items concerning choices that assessed one’s attitude toward favoring close others (e.g., family members, friends) at the cost of the greatest expected welfare. Agent-Centered Permissions dilemmas are composed of two items reflecting choices that assess one’s attitude toward improving others' welfare at a cost to one’s own interests. For example, whether to donate or keep the money for one’s own personal use. Personal Rights dilemmas are composed of two items concerning choices that substantially affected the interests of other people, and in which the best overall outcome could only be produced by violating an individual’s personal rights.

**Table 2. Semi-structured Interview Part B: Contextualization of Moral Reasoning**

| Domain | Follow up Questions |
| --- | --- |
| Values | Which is the most important value you try to live by? |
| Rule compliance | Would you break a rule to achieve a goal? |
